# ﻿Species of the genus *Laureola* (Isopoda, Armadillidae), with an identification key to world species

**DOI:** 10.3897/zookeys.1266.174151

**Published:** 2026-01-16

**Authors:** Zili Zong, Yutao Wang, Chao Jiang, Weichun Li

**Affiliations:** 1 College of Agronomy, Jiangxi Agricultural University, Nanchang 330045, China Jiangxi Agricultural University Nanchang China; 2 Haikou, China Unaffiliated Haikou China; 3 State Key Laboratory for Quality Ensurance and Sustainable Use of Dao-di Herbs, National Resource Center for Chinese Materia Medica, China Academy of Chinese Medical Sciences, Beijing 100700, China China Academy of Chinese Medical Sciences Beijing China

**Keywords:** Armadillidae, Hainan Island, morphology, new species, Oniscidea, taxonomy, woodlice

## Abstract

The genus *Laureola* Barnard, 1960 comprises twelve species distributed across the Afrotropical, Australian, and Oriental regions. In this study, we describe *Laureola volamuca* Zong, Wang, Jiang & Li, **sp. nov.**, the second species of the genus recorded in China, which was collected from Hainan Island. The habitus of the adult and male appendages of the new species are illustrated. An identification key to the world species of the genus is provided.

## ﻿Introduction

The genus *Laureola* Barnard, 1960 (Oniscidea, Armadillidae) was established with *Laureola paucispinosa* Barnard, 1949 designated as the type species (Barnard 1960a). It currently comprises twelve species distributed across the Afrotropical, Australian and Oriental regions (Barnard 1960a; Vandel 1973a; Ferrara and Taiti 1979; Schmalfuss and Ferrara 1983; Kwon et al. 1992; Li and Wang 2022). Prior to this study, only one species had been recorded from China (Li and Wang 2022). Here, we describe a second Chinese species of the genus based on specimens collected from Hainan Island, and provide a key to all *Laureola* species.

## ﻿Material and methods

Specimens were collected with fine forceps, preserved in 95% ethanol, and subsequently deposited in the Insect Museum, Jiangxi Agricultural University, Nanchang, China (JXAUM).

Appendages were dissected and mounted on microscope slides in neutral balsam. Images were captured with a Zeiss AxioCam Icc 5 camera attached to a Zeiss Stereo Discovery V12 microscope. Line drawings were drawn digitally using Adobe Photoshop (version 2024). The preparation of the identification key to world species of the genus *Laureola* was based on the studied specimens and the relevant literature (Barnard 1949, 1956, 1960a, 1960b; Vandel 1973b; Schmalfuss and Ferrara 1983; Kwon et al. 1992; Li and Wang 2022).

## ﻿Taxonomy

### ﻿Genus Laureola

Taxon classificationAnimaliaIsopodaArmadillidae

Barnard, 1960

FA53DC60-A4D5-52B4-8C15-18C99CD9EC61

Laureola Barnard, 1960a: 53.
Paralaureola Vandel, 1973a: 142 (nomen nudum).
Praelaureola Vandel, 1973b: 150.

#### Type species.

*Akermania paucispinosa* Barnard, 1949, by original designation.

#### Diagnosis.

Cephalon with a well-developed frontal shield. Body elliptic, bearing long spines or spiniform tubercles on dorsum. Pereonite 1 with bluntly round epimera, while pereonites 2−7 possess pointed epimera; ventral lobes present on pereonites 1, 2, and often 3. Pereopods elongated and fragile. Telson triangular except *L. canberrensis* (Vandel, 1973) and *L. dubia* Schmalfuss & Ferrara, 1983, which display rectangular distal portions. Uropod protopod typically triangular; exopod well-developed. Epimera of pleonites 3–5 with acutely pointed apical tips. All pleopodal exopods equipped with pleopodal lungs (see also Schmalfuss and Ferrara 1983).

#### Distribution.

Afrotropical, Australian, and Oriental regions.

### ﻿ Laureola volamuca

Taxon classificationAnimaliaIsopodaArmadillidae

Zong, Wang, Jiang & Li sp. nov.

CC4C56C5-DA6C-5160-8D54-D1BDEE4FDEEC

https://zoobank.org/A81D4045-02CD-46AE-8E93-9110055261DC

#### Type materials.

***Holotype*** • ♂, China, Hainan Province, Qiongzhong Li and Miao Autonomous County, Limu Mountain National Forest Park (19.2876°N, 109.7992°E), 1350 m, 6 November 2023, Yutao Wang leg., prep. slide nos. L23107, L24054–L24055. ***Paratypes***: 26 ♂♂, 13 ♀♀, same data as holotype.

#### Diagnosis.

Cephalon with frontal shield gently arched medially; pereopod 7 bearing a triangular process at distal tip of basis on sternal face; telson with a round distal tip.

#### Description.

**Figure 1.**
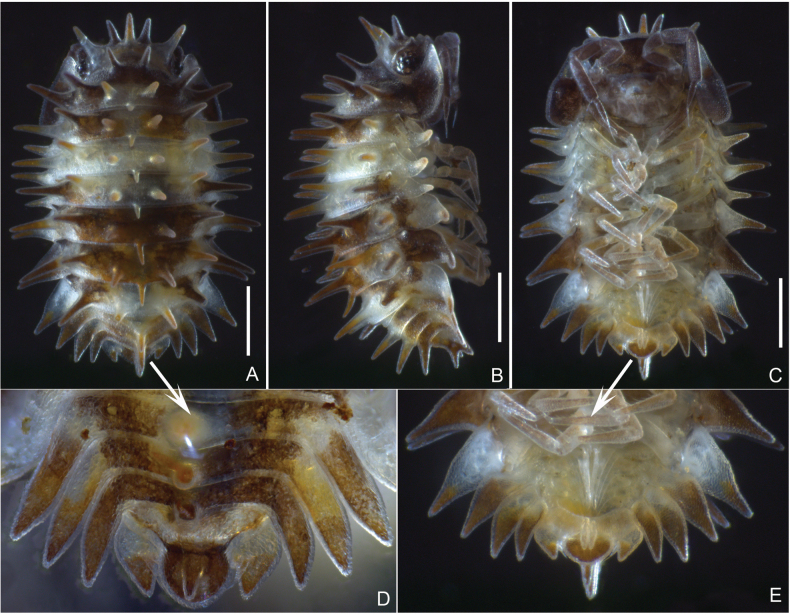
*Laureola volamuca* Zong, Wang, Jiang & Li, sp. nov. **A.** Male adult, dorsal view **B.** Ditto, lateral view **C.** Ditto, ventral view **D.** Pleonites and telson, dorsal view **E.** Ditto, ventral view. Scale bars: 0.5 mm.

***External morphology of adult*** (Fig. 1). Maximum body length 3.5 mm in males, 3.8 mm in females. Body elliptic, unable to roll up into endoantennal conglobation. Dorsal surface yellowish brown, with extensive yellowish-white areas on pereonites 3, 4, and 7. Dorsum bearing long spines arranged as follows: cephalon with 4+1; pereonite 1 with 2+7; pereonites 2–6 each with 7; pereonite 7 with 4; pleonites 3–5 and telson each with single median spine.

**Figure 2.**
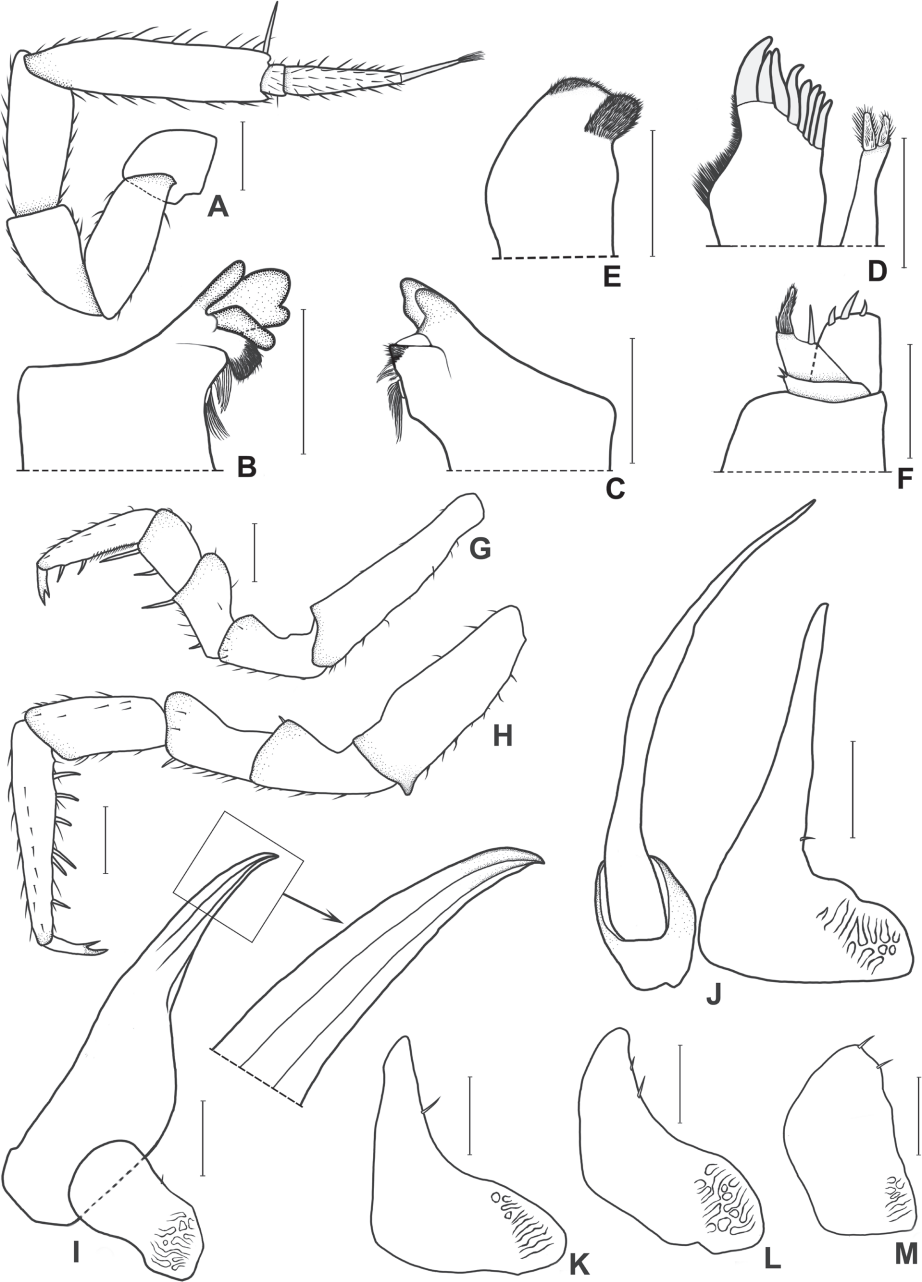
*Laureola volamuca* Zong, Wang, Jiang & Li, sp. nov. **A.** Antenna **B.** Left mandible **C.** Right mandible **D.** Maxillule **E.** Maxilla **F.** Maxilliped **G.** Pereopod 1 **H.** Pereopod 7 **I.** Pleopod 1 **J.** Pleopod 2 **K.** Pleopod 3 exopod **L.** Pleopod 4 exopod **M.** Pleopod 5 exopod. Scale bars: 0.1 mm.

***Cephalon*** (Figs 1A–C, 2A–F). Frontal shield not protruding above vertex, gently arched medially. Eye composed of 10–12 ommatidia. Antennule with three articles, distal article with eight to ten superimposed aesthetascs near apex. Antenna bearing long spine at distal apex of fifth peduncular article; flagellum with second article (including apical organ) approximately eight times as long as first article, apical organ thin and long. Left mandible with three-toothed incisor, five penicils between hairy lobe and molar process; right mandible with two-toothed incisor, four penicils between hairy lobe and molar process. Maxillule with two unequal penicils on distal margin of inner lobe; outer lobe with eight apical teeth of various sizes. Maxilla bilobate apically, inner lobe smaller and bearing more setae than outer lobe. Maxilliped endite rectangular, apex with three setae; palp three-articled, basal article with two small setae, medial article with one large seta at inner distal angle of distal margin, distal article densely covered with hairy setae.

***Pereon*** (Figs 1A−C, 2G, H). Pereonite 1 with bluntly round epimera; pereonites 2−7 with pointed epimera. Ventral lobes present on pereonites 1–3. Pereopods without apparent sexual specializations; pereopod 1 basis thin and long, ischium concave on rostral face; pereopod 7 basis bearing small triangular process distally on sternal face, ischium concave on rostral face.

***Pleon*** (Fig. 1). Pleonites 3–5 with elongate, spine-shaped epimera. Telson broad basally, narrowed towards middle, distal half elliptic. Uropod protopod nearly triangular; exopod inserted near medial margin, approximately one-third length of protopod, not extending to its apex.

***Male pleopods*** (Fig. 2I−M). Pleopod 1 exopod small, twice as wide as long, distal part round; endopod with broad basal portion, tapering to pointed tip, apical portion directed outwards. Pleopod 2 exopod broad in basal one-third, distal two-thirds thin and long, outer margin strongly concave; endopod thin and long, surpassing exopod. Pleopods 3−4 exopods subtriangular, outer margin concave. Pleopod 5 exopod subquadrangular, outer margin nearly straight.

#### Etymology.

The specific name is from the Latin “*vola*” (meaning “fly”) and “*mucus*” (meaning “nasal mucus”), referring to the species’ distinctive jumping locomotion, reminiscent of flying nasal mucus. We suggest the Chinese common name as “鼻屎精灵刺卷虫”.

#### Distribution.

China (Hainan).

#### Natural history.

This species was collected in the ecotone between coniferous and broad-leaved forests. It forages diurnally on stones covered with algae and moss. At night, it seeks shelter under the webs of jumping spiders and also forages on remnants of prey left within these webs. The species exhibits a characteristic jumping escape response when disturbed (see Suppl. material 1: videos 1, 2).

#### Remarks.

The new species is similar to *Laureola leucocephala* Li & Wang, 2022, but it can be distinguished by the following characters: cephalon with frontal shield not protruding above the vertex and is gently arched medially; the basis of pereopod 7 bears a triangular process distally on the sternal face; the telson has a rounded distal tip; and the uropod exopod does not reach the tip of the protopod. In *L. leucocephala*, the frontal shield distinctly protrudes above the vertex and bears a broad median triangular process; the basis of pereopod 7 lacks any projection; the telson tapers to a pointed distal tip; and the uropod exopod exceeds the tip of the protopod.

##### ﻿Key to the species of the genus *Laureola* Barnard, 1960

**Table:** 

1	Pleonites 3 and 4 each with one spine	**2**
–	Pleonites 3 and 4 each with two or three spines	**8**
2	Uropod exopod extending beyond apex of protopod	**3**
–	Uropod exopod not reaching apex of protopod	**6**
3	Pereonite 7 with five or seven spines	**4**
–	Pereonite 7 with more than seven spines	**5**
4	Pereonites 2–7 each with five spines	***L. indica***
–	Pereonites 2–6 each with nine spines, pereonite 7 with seven spines	***L. vietnamensis***
5	Pereonites 2–6 with nine spines, pereonite 7 with eight spines	***L. leucocephala***
–	Pereonites 2–4 each with eleven spines, pereonites 5–7 each with nine spines	***L. miacantha***
6	Telson with a round distal portion	***L. volamuca* sp. nov.**
–	Telson with a triangular distal portion	**7**
7	Cephalon with arranging in two rows of six spines	***L. longispina***
–	Cephalon with arranging in three rows of eight spines	***L. paucispinosa***
8	Pleonites 3 and 4 each with two spines	**9**
–	Pleonites 3 and 4 each with three spines	**11**
9	Telson with a triangular distal portion	***L. silvatica***
–	Telson with a rectangular distal portion	**10**
10	Telson with two spines, distal apex without projection	***L. canberrensis***
–	Telson without two spines, distal apex with a triangular projection on each lateral margin	***L. dubia***
11	Telson without long spines, some individuals with one small spine	***L. rubicunda***
–	Telson with distinctively long spines	**12**
12	Telson with one spine	***L. hiatus***
–	Telson with two spines	***L. bivomer***

## Supplementary Material

XML Treatment for Laureola

XML Treatment for Laureola volamuca

## ﻿Additional information

### Use of AI

No use of AI was reported.

### Author ORCIDs

Zili Zong https://orcid.org/0009-0000-2793-2420

Chao Jiang https://orcid.org/0000-0003-1841-1169

Weichun Li https://orcid.org/0000-0003-0154-861X

### Data availability

All of the data that support the findings of this study are available in the main text or Supplementary Information.

## Supplementary materials

Supplementary material 1 Videos 1, 2 Data type zip Explanation note Video recordings depicting the escape behaviour of *Laureola volamuca* Zong, Wang, Jiang & Li, sp. nov. The species exhibits a characteristic forward-jumping motion when disturbed. https://binary.pensoft.net/file/1513531This dataset is made available under the Open Database License (http://opendatacommons.org/licenses/odbl/1.0/). The Open Database License (ODbL) is a license agreement intended to allow users to freely share, modify, and use this Dataset while maintaining this same freedom for others, provided that the original source and author(s) are credited.Zili Zong, Yutao Wang, Chao Jiang, Weichun Li
